# Pharmacological Manipulation of Corticotropin-Releasing Factor Receptors in the Anterior and Posterior Subregions of the Insular Cortex Differently Affects Anxiety-Like Behaviors in the Elevated Plus Maze in Rats

**DOI:** 10.1155/2024/8322844

**Published:** 2024-01-31

**Authors:** Lucas M. Lopes, Lilian L. Reis-Silva, Bruno Rodrigues, Carlos C. Crestani

**Affiliations:** ^1^Laboratory of Pharmacology, School of Pharmaceutical Sciences, São Paulo State University (UNESP), Araraquara, São Paulo, Brazil; ^2^Department of Adapted Physical Activity, Faculty of Physical Education, University of Campinas (UNICAMP), Campinas, São Paulo, Brazil

## Abstract

Neuroimaging data in humans and neurobiological studies in rodents have suggested an involvement of the insular cortex (IC) in anxiety manifestations. However, the local neurochemical mechanisms involved are still poorly understood. Corticotropin-releasing factor (CRF) neurotransmission has been described as a prominent neurochemical mechanism involved in the expression of anxiety-like behaviors, but the brain sites related are poorly understood. Additionally, several findings indicate that control of physiological and behavioral responses by the IC occurs in a site-specific manner along its rostrocaudal axis. Thus, this study is aimed at evaluating the effect of CRF receptor agonism and antagonism within the anterior and posterior subregions of the IC in controlling anxiety-related behaviors in the elevated plus maze (EPM). For this, independent groups (six groups) of animals received bilateral microinjections of vehicle, the selective CRF_1_ receptor antagonist CP376395, or CRF into either the anterior or posterior subregions of the IC. Ten minutes later, the behavior in the EPM was evaluated for five minutes. Treatment of the anterior IC with CP376395, but not with CRF, increased the time and number of entries into the open arms of the EPM. CRF, but not the CRF_1_ receptor antagonist, microinjected into the posterior IC also increased exploration of the EPM open arms. Taken together, these data indicate that CRFergic neurotransmission in the anterior IC is involved in the expression of anxiety-related behaviors in the EPM. This neurochemical mechanism does not seem to be activated within the posterior IC during exposure to the EPM, but the effects caused by CRF microinjection indicate that activation of CRF receptors in this IC subregion might evoke anxiolytic-like effects.

## 1. Introduction

The insular cortex (IC) is a cortical brain structure that presents anatomical integration with cortical and subcortical regions and, through these connections, participates in the control of sensory, emotional, physiological, motivational, and cognitive functions [1–3]. Accordingly, clinical and preclinical studies have provided evidence of a prominent role of the IC in anxiety manifestations [2, 4, 5]. For instance, neuroimaging studies identified that patients diagnosed with anxiety disorders presented increased IC activity [6–8] and anxiolytic effects of behavioral and pharmacological therapies were followed by altered IC activity [5, 6, 9]. Experiments in rodents have also indicated a role of the IC in the expression of anxiety-like behaviors related to conditioned and unconditioned stimuli [10–15].

Studies in rodents have indicated that control of anxiety-related behaviors seems to occur in a site-specific manner along the rostrocaudal axis of the IC [12–14, 16]. For instance, it was reported that excitotoxic lesion or antagonism of non-NMDA glutamatergic receptors in the anterior subregion of the IC caused anxiolytic-like effects in the elevated plus maze (EPM) [13, 14], whereas non-NMDA receptor antagonism in the posterior IC caused anxiogenic-like response [13]. Despite the evidence stated above, the local neurochemical mechanisms involved in the control of behavioral responses related to anxiety by the different sites along the rostrocaudal axis of the IC are still poorly understood.

Corticotropin-releasing factor (CRF) neurotransmission was identified throughout the brain and has been implicated in the expression of defensive and anxiety-like behaviors [17–20]. Accordingly, circuits containing the CRFergic system play an important role in the pathophysiology of anxiety [19–22]. In this sense, most studies have shown that activation of CRF_1_ receptors is involved in the expression of anxiogenic-like responses [17, 18, 20, 23, 24]. However, control of behavioral responses by CRF_1_ receptors seems to be site-specific in the brain, and anxiolytic-like effects have also been related to activation of CRF_1_ receptors in some regions, such as the frontal cortex [25, 26]. Although evidence obtained using *knockout* mice for the CRF_2_ receptor indicates an anxiolytic role of this receptor [27], the data using pharmacological approach are inconsistent, so the role of CRF_2_ receptors is not completely understood [17, 18, 20].

CRF has been identified as the main endogenous ligand of CRF receptors in the cerebral cortex; and the cerebral cortex, including IC, expresses predominantly the CRF_1_ receptor [17, 28–30]. Indeed, a recent study identified the presence of CRF_1_ receptor mRNA, but not CRF_2_ receptor mRNA, within the IC [31]. Additionally, immunoreactive fibers for CRF were identified [32], thus suggesting that this neurochemical transmitter is released within the IC from inputs coming from other brain regions. However, CRF mRNA was not identified [31], which indicates that CRF is not synthesized by local IC neurons. CRFergic neurotransmission was described as a prominent neurochemical mechanism mediating the control of emotional responses by the IC [31]. However, to the best of our knowledge, a systematic analysis of the role of CRFergic mechanisms controlling anxiety-like behaviors in specific sites along the rostrocaudal axis of the IC has never been reported. This latter aspect is relevant considering evidence that control of defensive behaviors by brain CRF circuits might be site-specific [17, 22]. Therefore, we investigated the effects of CRF receptor agonism and antagonism in the anterior and posterior subregions of the IC in the expression of anxiety-like behaviors in the EPM.

## 2. Materials and Methods

### 2.1. Animals

Fifty-one male Wistar rats with body weight between 240 and 250 g (60 days old) provided by the Central Animal Facility of the São Paulo State University (UNESP) (Botucatu, São Paulo, Brazil) were used in the present study. All rats were acclimated in the Animal Facility of the Laboratory of Pharmacology of the School of Pharmaceutical Sciences, UNESP (Araraquara, São Paulo, Brazil), for a period of at least seven days before beginning the experimental procedures. The animals had free access to food and water throughout the protocol (except during the brief trial periods) and were kept in temperature-controlled rooms (temperature 23 ± 2°C and humidity 50 ± 10%) with 12 h dark-light cycle (lights on between 7 p.m. and 7 a.m.) in collective plastic home cages. All experimental procedures were approved by the Ethical Committee for Use of Animals of the School of Pharmaceutical Sciences, UNESP (approval # 20/2021).

### 2.2. Drugs and Solutions

The selective CRF_1_ receptor antagonist CP376395 (Tocris, Westwoods Business Park, Ellisville, Missouri, USA), corticotropin-releasing factor (CRF) (Sigma-Aldrich, St. Louis, Missouri, USA), and urethane (Sigma-Aldrich) were dissolved in saline (0.9% NaCl). The veterinary pentabiotic (Fort Dodge, Campinas, SP, Brazil), isoflurane (Cristrália, Itapira, São Paulo, Brazil), and the nonsteroidal anti-inflammatory flunixin meglumine (Banamine, Schering-Plough, Cotia, SP, Brazil) were used as supplied by the manufacturers.

### 2.3. Surgical Preparation

The animals underwent inhalation anesthesia with isoflurane (2.5%) using a low-flow vaporizer (Bonther, Ribeirão Preto, São Paulo, Brazil). After trichotomy, the rat's head was immobilized in a stereotaxic apparatus (Stoelting, Wood Dale, Illinois, USA). For local anesthesia and reduction of bleeding, 0.3 mL of lidocaine solution with vasoconstrictor (2%lidocaine + 3%phenylephrine, Harvey Química Farmacêutica Ind. e Comércio Ltda, Catanduva, São Paulo, Brazil) was injected subcutaneously. Then, the skull was exposed through a skin incision, and the region was cleaned using saline solution and 10% hydrogen peroxide. All coordinates for the implantation of guide cannulas directed to either the anterior or posterior IC were based on parameters obtained from the atlas of the rat brain by Paxinos and Watson [33]. Stereotaxic coordinates were anteroposterior, +10.5 mm (anterior IC) or +6.9 mm (posterior IC) in relation to interaural; lateral, +3.8 mm (anterior IC) or +6.2 mm (posterior IC) from the middle suture; and vertical, -4.5 mm (anterior IC) or -6.0 mm (posterior IC) in relation to the skull.

After positioning the stainless steel cannula (26 G, 12 mm), small holes were made in the skull using a dental drill, through which the cannulas were bilaterally introduced. The cannulas were fixed to the skull using a self-curing acrylic resin (Simplex, DFL, Ind. Com., Rio de Janeiro, Rio de Janeiro, Brazil), and small stainless steel screws were previously implanted in the skull for resin fixation. A 0.2 mm diameter mandrel was introduced into the cannulas to avoid obstruction during the recovery period. As a prophylactic approach, after the end of the surgical procedure, the animals received 0.2 mL of veterinary pentabiotic (Fontoura-Wyeth, São Paulo, Brazil) intramuscularly (560 mg/mL/kg) and 0.3 mL of the nonsteroidal anti-inflammatory flunixin meglumine subcutaneously (0.5 mg/mL/kg). The animals were kept in recovery for at least four days after the surgical procedure.

### 2.4. Experimental Design

Independent sets of animals underwent stereotaxic surgery for implantation of bilateral guide cannulas directed to either the anterior or posterior subregions of the IC and then were kept in recovery for at least four days. Definitions of the IC rostrocaudal subregions were based on previous studies [34–39]. On the test day, the animals were taken to the trial room at least 60 min before the start of the experiment for habituation to the room conditions. All tests were conducted during the light phase of the light/dark cycle (11 and 15 lux on the floor of closed and open arms, respectively).

The animals were randomly divided into six experimental groups: (i) CP376395/anterior IC, which received bilateral microinjection of the selective CRF_1_ receptor antagonist CP376395 (5 nmol/100 nL) into the anterior subregion of the IC (*n* = 8); (ii) CRF/anterior IC, which received bilateral microinjection of CRF (CRF receptor agonist) (0.07 nmol/100 nL) into the anterior subregion of the IC (*n* = 9); (iii) vehicle/anterior IC, which received bilateral microinjection of vehicle (saline, 100 nL) into the anterior subregion of the IC (*n* = 10); (iv) CP376395/posterior IC, which received bilateral microinjection of the selective CRF_1_ receptor antagonist CP376395 (5 nmol/100 nL) into the posterior subregion of the IC (*n* = 8); (v) CRF/posterior IC, which received bilateral microinjection of CRF (0.07 nmol/100 nL) into the posterior subregion of the IC (*n* = 9); and (vi) vehicle/anterior IC, which received bilateral microinjection of vehicle (saline, 100 nL) into the posterior IC (*n* = 7). Ten minutes after IC treatment, the animals were individually placed on the central square always facing the open arm of the EPM and were allowed to freely explore the apparatus for 5 minutes. Drug doses and treatment protocol (i.e., 10 min before a test) were based on a previous study from our group [40].

### 2.5. Intrabrain Drug Microinjection

The injection needles (33 G, Small Parts, Miami Lakes, Florida, USA) used for intrabrain microinjection of the drugs were 1 mm longer than the guide cannulas previously fixed to the skull and were connected to a 2 *μ*L syringe (7002-KH, Hamilton Co., Reno, NV, USA) through a polyethylene tube (PE-10) (Clay Adams, Parsippany, NJ, USA). The drugs were injected in a volume of 100 nL/side [11, 16, 34].

### 2.6. Behavioral Analysis in the Elevated Plus Maze (EPM)

The EPM was used to assess the effects of pharmacological treatments of the IC on anxiety-related behaviors [41–44]. The apparatus used for the test consisted of two open arms and two closed arms, 50 cm long and 10 cm wide each, joined perpendicularly and elevated 50 cm from the ground. The closed arms had 40 cm high walls that surrounded them, and the open arms had a 1 cm acrylic side border to prevent the animals from falling out of the apparatus.

Rodents avoid open arms, and anxiolytic drugs typically increase exploration of open arms without interfering with the number of entries into closed arms [45]. Thus, in the present study, the number of entries into the open and closed arms and the time spent in the open arms were quantified [43, 44, 46]. In the interval between tests of each animal, the maze was cleaned with an alcohol solution (20%). Animal behaviors were recorded for a period of 5 minutes and automatically analyzed using the Anymaze software (Stoelting, Wood Dale, IL, USA).

### 2.7. Histological Determination of Microinjection Sites in the Brain

At the end of each experiment, the animals were anesthetized with urethane (250 mg/mL/200 g body weight, i.p.) and 100 nL of Evans blue dye (1%) was bilaterally injected to determine the microinjection sites in the brain. Then, the rats were perfused, and the brain was removed and postfixed in 10% formalin for at least 48 h at 4°C. Subsequently, the brain was sectioned into 40 *μ*m thick frontal cuts using a cryostat (CM1900, Leica, Wetzlar, Germany) for analysis of the microinjection sites. The placement of the microinjection needles was determined in a light microscope according to the rat brain atlas of Paxinos and Watson [33].

### 2.8. Statistical Analysis

Data were expressed as mean ± standard error of the mean (SEM). All data were analyzed using the software GraphPad Prism version 7.0 (GraphPad Software Inc., La Jolla, CA, USA). All behavioral parameters in the EPM were compared using one-way ANOVA. When ANOVA indicated statistical differences, the Bonferroni *post hoc* test was used to assess specific differences between the experimental groups. Results with *P* < 0.05 were considered statistically significant.

## 3. Results

**Figure 1:**
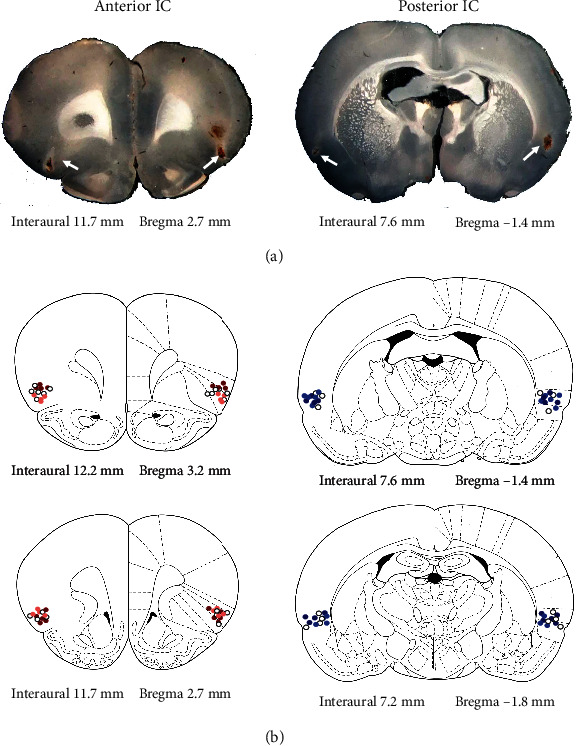
Microinjection sites into the anterior and posterior subregions of the IC. (a) Photomicrographs of coronal brain sections depicting the microinjection sites of representative animals in the anterior and posterior subregions of the IC. Arrows indicate the microinjection sites. (b) Diagrammatic representations modified from the rat brain atlas of Paxinos and Watson [58] indicating the microinjection sites into the anterior (left images) and posterior (right images) subregions of the IC of vehicle (white circles), the selective CRF_1_ receptor antagonist CP376395 (anterior IC, orange circles; posterior IC, light blue circles), and CRF (anterior IC, red circles; posterior IC, dark blue circles) of all animals used in the present study. The numbers indicate the interaural and bregma distances.

Diagrammatic representations of coronal sections based on the rat brain atlas of Paxinos and Watson [33] showing microinjection sites into the anterior and posterior subregions of the IC of all animals included in the present study are presented in Figure 1. Figure 1 also shows photomicrographs of coronal brain sections of representative animals showing the microinjection sites into the anterior and posterior IC.

### 3.1. Effect of CRF and CP376395 Microinjection into the Anterior IC on Anxiety-Like Behaviors in the EPM

**Figure 2:**
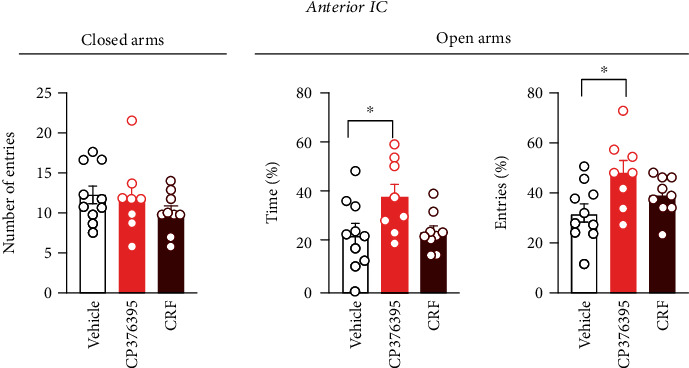
Role of CRFergic neurotransmission in the anterior IC controlling anxiety-like behaviors in the EPM. Effect of bilateral microinjection of vehicle (saline, 100 nL, *n* = 10), the selective CRF_1_ receptor antagonist CP376395 (5 nmol/100 nL, *n* = 8), or CRF (0.1 nmol/100 nL, *n* = 9) into the anterior subregion of the IC on the exploration of open and closed arms of the EPM. Treatment of the anterior IC was performed 10 min before the EPM exposure. Columns represent the mean ± SEM. ^∗^*P* < 0.05 in relation to the vehicle group. One-way ANOVA followed by the Bonferroni *post hoc* test.

Analysis of the percentage of time (*F*_(2, 24)_ = 3.7, *P* = 0.0384) and entries (*F*_(2, 24)_ = 4.429, *P* = 0.0231) into the open arms of the EPM indicated a difference between the experimental groups (Figure 2). *Post hoc* analysis revealed that antagonism of CRF_1_ receptors in the anterior IC via local microinjection of CP376395 (*n* = 8) increased the time (*P* = 0.0498) and number of entries (*P* = 0.0174) into the open arms, when compared to animals that received bilateral microinjection of vehicle into the IC (*n* = 10) (Figure 2). Analysis did not suggest effect on time (*P* > 0.05) and number of entries (*P* > 0.05) into the open arms in animals that received CRF (*n* = 9) into the anterior IC (Figure 2). Analysis of the number of entries into the closed arms of the EPM did not indicate differences between the experimental groups (*F*_(2, 24)_ = 1.04, *P* = 0.3689) (Figure 2).

### 3.2. Effect of CRF and CP376395 Microinjection into the Posterior IC on Anxiety-Like Behaviors in the EPM

**Figure 3:**
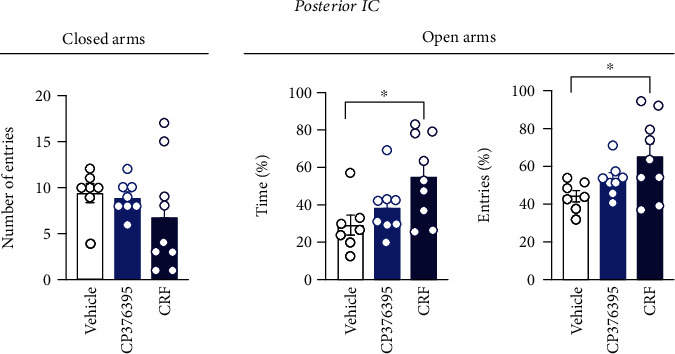
Role of CRFergic neurotransmission in the posterior IC controlling anxiety-like behaviors in the EPM. Effect of bilateral microinjection of vehicle (saline, 100 nL, *n* = 7), the selective CRF_1_ receptor antagonist CP376395 (5 nmol/100 nL, *n* = 8), or CRF (0.1 nmol/100 nL, *n* = 9) into the posterior subregion of the IC on the exploration of open and closed arms of the EPM. Treatment of the posterior IC was performed 10 min before the EPM exposure. Columns represent the mean ± SEM. ^∗^*P* < 0.05 in relation to the vehicle group. One-way ANOVA followed by the Bonferroni *post hoc* test.

Analysis of the percentage of time (*F*_(2, 21)_ = 4.29, *P* = 0.0272) and entries (*F*_(2, 21)_ = 4.13, *P* = 0.0307) into the open arms of the EPM indicated a difference between the experimental groups (Figure 3). *Post hoc* analysis revealed that activation of CRF receptors within posterior IC by local microinjection of CRF (*n* = 9) increased the time (*P* = 0.0247) and number of entries (*P* = 0.0258) into the open arms, when compared to animals that received local bilateral microinjection of vehicle (*n* = 7) (Figure 3). Analysis did not indicate effect on either time (*P* > 0.05) or number of entries (*P* > 0.05) into the EPM open arms in animals treated with the selective CRF_1_ receptor antagonist CP376395 into the posterior IC (*n* = 8) (Figure 3). Analysis of the number of entries into the EPM closed arms did not indicate differences between the experimental groups (*F*_(2, 21)_ = 0.98, *P* = 0.3693) (Figure 3).

## 4. Discussion

The results reported in the present study provide the first evidence that control of anxiety-like behaviors by local CRFergic neurotransmission is site-specific along the rostrocaudal axis of the IC. Indeed, our data suggest that activation of CRF_1_ receptors in the anterior subregion of the IC is involved in the expression of anxiety-related behaviors in the EPM. Conversely, activation of CRFergic receptors within the posterior subregion of the IC might evoke anxiolytic-like effects.

Neurotransmission of the CRF system acting via CRF_1_ receptors has been described as an important brain neurochemical mechanism related to the expression of anxiogenic-like responses [18–20, 23, 24, 47]. In this sense, our data provide new evidence by indicating the anterior subregion of the IC as a prominent brain site related to the role of CRFergic neurotransmission in the expression of anxiety behaviors. The findings obtained in the present study are in line with previous evidence that indicated a relevant role of the anterior IC in anxiety. Indeed, neuroimaging studies in humans have indicated the anterior subregion as the IC site involved in the expression of emotions [48–50]. Studies in rodents have also identified that excitotoxic lesions or antagonism of non-NMDA glutamate receptors within the anterior IC caused anxiolytic-like effects in the EPM [13, 14], whereas chemogenetic activation caused anxiogenic-like effects in animals previously subjected to restraint stress [51]. In this sense, the data obtained in the present study provide new evidence indicating that CRFergic neurotransmission is an important local neurochemical mechanism involved in the control of anxiety-related behaviors by the anterior IC. In this sense, the results reported here also provide advances in the understanding of neuroimaging studies performed in humans that indicated increased activity in the IC of patients diagnosed with anxiety disorders [6–8]. However, it should be noted that the anterior IC does not seem to be involved in all manifestations of anxiety responses, since the chemical inhibition of this region did not affect contextual and auditory fear conditioning [12].

Regarding the posterior subregion of the IC, we observed that local pharmacological treatment with the CRF_1_ receptor antagonist did not cause behavioral changes in the EPM. However, bilateral microinjection of CRF increased open-arm exploration, thus indicating an anxiolytic-like effect. The lack of effect of CRF_1_ receptor antagonism suggests that CRFergic neurotransmission is not recruited within the posterior IC during EPM exposure. Our findings corroborate previous evidence that excitotoxic lesions in the posterior IC did not affect behavioral responses in the EPM [14], though this IC subregion seems to play a role in the expression of freezing response to fear conditioning [12, 52]. However, the change in open-arm exploration after pharmacological activation of CRFergic receptors through local microinjection of CRF indicates that this neurochemical mechanism can generate anxiolytic effects in the posterior IC. Thus, although our data indicate that CRFergic neurotransmission within the posterior IC is not recruited for the expression of innate anxiety, it may be involved in the modulation of anxiogenic responses caused by stimuli that activate CRFergic mechanisms in the cerebral cortex. In this sense, brain CRFergic mechanisms are activated during emotional stress in several brain regions, including the cerebral cortex [47, 53]. Thus, further studies are needed to explore a possible modulatory role of CRFergic neurotransmission within the posterior IC in stress-evoked anxiogenic responses.

Taken together, the findings reported here indicate an opposing role of CRFergic neurotransmission in the anterior (anxiogenic role) and posterior (anxiolytic role) subregions of the IC controlling behaviors in the EPM. The findings of the present study are similar in relation to those obtained previously following antagonism of non-NMDA glutamatergic receptors in the IC [13]. Indeed, results obtained in this previous study evidenced an involvement of glutamatergic neurotransmission within the anterior IC in the expression of anxiety-like behaviors in the EPM, whereas an anxiolytic role was identified in the posterior IC [13]. This opposite control seems to be related to different connections of the IC subregions. Indeed, a study that systematically evaluated connections from different sites along the rostrocaudal axis of the IC identified specific projections from the anterior versus posterior subregions [39]. Accordingly, it was reported that the role of the anterior IC, but not posterior IC, in behavioral control in the EPM seems to be related to projections to the basolateral amygdala (BLA) [14], whereas the control of anxiety by the posterior IC seems to be mediated, at least partially, by connection with the central nucleus of the amygdala (CeA) [54].

The opposite role of the CRFergic neurotransmission in the anterior and posterior IC might also be mediated by different effects on local neuronal activation. In this sense, it was reported that the expression of anxiety responses in the EPM is related to an increase in neuronal activity in the posterior IC [54], so an enhanced open-arm exploration was observed following the silencing of neurons within this IC subregion. Therefore, control by CRF neurotransmission in the anterior IC could occur via activation of local pyramidal neurons projecting to the BLA (see discussion above), which in turn would result in an anxiogenic effect. On the other hand, activation of CRF receptors within the posterior IC could inhibit local CeA-projecting neurons, thus causing anxiolytic effects. This idea is further supported by previous results indicating a deactivation of posterior IC neurons during stressful events [34]. However, precluding this hypothesis, a recent study identified that CRF acting via the CRF_1_ receptor depolarizes pyramidal neurons within the posterior IC [31]. Therefore, further studies are necessary to explore the local mechanisms and circuitry related to the opposite role of the IC CRFergic neurotransmission in the control of anxiety-like behaviors.

Another mechanism that may explain the opposite effects of CRFergic neurotransmission within the anterior and posterior IC is an eventual influence on the internal information flow within the IC. In this sense, studies have provided evidence of a flow of interoceptive information from the posterior to the anterior subregion, so that the anterior IC would be responsible for integrating the various kinds of information that reaches this cerebral structure and evoking emotional responses [50, 55, 56]. Accordingly, a recent study identified a role of the posterior IC in anxiogenic response caused by cardiac activation [57]. Thus, a possibility is that control by the CRFergic neurotransmission in the posterior IC is related to an influence on internal circuits within the IC, which would preclude the activation of pyramidal neurons in the anterior IC involved in the expression of anxiety.

A limitation of the present study was the use of only one behavioral test (i.e., EPM) for evaluation of the IC CRF neurotransmission involvement in the expression and modulation of anxiety-like behaviors. However, as stated above, our findings are in line with previous studies that evaluated the role of the IC in anxiety-like behaviors using several tests [14]. Therefore, although it constitutes a limitation, we do not expect that the use of only one behavioral test affected the interpretations and conclusions of the study.

In summary, the results reported in the present study indicate that the CRFergic neurotransmission in the IC is involved in the control of anxiety-like behaviors and this regulation occurs in a site-specific manner along the rostrocaudal axis of the IC. Indeed, the results suggest an involvement of CRF_1_ receptors in the anterior IC in the expression of anxiety-related behaviors in the EPM. Although the CRFergic neurotransmission seems not to be recruited within the posterior IC during exposure to the EPM, our findings indicate that activation of CRF receptors in this brain site might evoke anxiolytic-like effects. Therefore, further studies are needed to clarify the eventual role of the CRFergic neurotransmission within the posterior IC in behavioral changes caused by stimuli in which this neurochemical mechanism is recruited in cortical structures, such as stressful events.

## Data Availability

The data used to support the findings of this study are available from the corresponding author upon request.

## Abbreviations

BLA: Basolateral amygdala
CeA: Central nucleus of the amygdala
CRF: Corticotropin-releasing factor
EPM: Elevated plus maze
IC: Insular cortex.
